# War Neuroses

**Published:** 1918-08

**Authors:** 


### WAR NEUROSES

The discussion on " War Neuroses " at the Meeting of the Research Society, June 29, was participated in by a number of French and British neurologists who related their experiences in dealing with the nervous cases that have occurred in the Allied Armies during the past four years. These men have had the opportunity of trying out the different methods of handling the war neuroses; and having had sufficient time to observe the end result of various plans of treatment, their conclusions are of great value.

An important fact brought out in the discussion was that the medical officer at the front is the man who is often responsible for many suggestions that tend to make chronic hospital patients of neurotic and fatigued soldiers, who, if properly treated, would never go further back from the line than the regimental hospital. It is therefore essential that regimental medical officers, as well as the other medical men with the American Expeditionary Forces, should be informed regarding the diagnosis and treatment of the psychoneuroses that may occur in soldiers.

In this symposium on " War Neuroses ", the British Army was represented by Major Foster Kennedy, R. A. M. C., F. R. S. Edin., who discussed " The Nature of Nervousness in Soldiers ", and by Lieut-Colonel Gordon Holmes, R. A. M. C., whose subject was " The Treatment and Management of Psychoneuroses as they Appear in a Combatant Army. " The French viewpoint was expressed by Professor Dupré, who discussed " The Emotions and the War " ; Professor G. Roussy, who dealt with the " Psychoneurological Disturbances Affecting the Limbs " ; Professor Laignel-Lavastine, whose subject was " Observations and Diagnosis and Treatment of War Neuroses " ; and Professor Marie, who talked on " Organic Causes of Psychic Disturbances ".

The Medical Department of the American Expeditionary Forces was represented by Lieut-Colonel Thomas Salmon, Chief Consultant in Psychoneurology, and Major J. H. W. Rhein, M. R. C., also connected with the Neurological Division. Lieut-Col. Salmon discussed " American Plans for Dealing with War Neuroses ", calling attention to what has been done, and what will be done, by the Division of Neurology of the American Expeditionary Forces ; and Major Rhein told of the methods that have been employed in the United States to eliminate neurotics and psychasthenics from the American Army.

About two hundred American physicians had the privilege of hearing the discussions on " War Neuroses ", but every medical officer with the American Expeditionary Forces will have the opportunity of reading in *War Medicine* the reports of this important discussion of the Research Society.

### No More Shell-Shock

There were some slight differences of opinions among the neurologists who participated in the discussion on " War Neuroses " at the meeting of the Research Society ; but all agreed that there is no place in medical nomenclature for " shell-shock ". At the beginning of the war, there were many medical articles published under the title of " shell-shock " which gave to medical men a wrong conception of the condition. The public was fed on a lot of misinformation, and very soon " shell-shock " was regarded among the laity as a new and mysterious condition resulting from the concussion incident to the use of high explosives in modern warfare. Soldiers who were supposed to be suffering from " shell-shock ", returning to their homes, or to base hospitals, were sought out by curiosity-seekers, and were given a lot of sympathy and attention, which only exaggerated their symptoms.

Soon it was found that many soldiers who were in positions to suffer the greatest concussion from heavy artillery or bursting shells were not affected. It was then realized that it was the men of neurotic type, and those who were excessively fatigued, who, when the Army surgeon made the snapshot diagnosis " shell-shock ", spent months in base or neurological hospitals because of the suggestion and the patient's misconception of his true condition. Then it was realized by the French and British neurologists that " shell-shock " in war was the same thing as the traumatic neuroses in civil life.

The term has been given up and the soldiers of the French and British Armies are now ashamed to admit that they have " shell-shock "; because, as one neurologist said, " the good soldier, except when excessively fatigued, does not have " shell-shock ", and then the symptoms are of short duration and subside after a brief rest ". The so-called " shell-shock " cases are now treated at the Front by the British and French Army surgeons, and they usually return to duty in a few days.

It is well for American medical officers to get the viewpoint of our French and British confreres who have had a large experience with the war neuroses, and under no circumstances to make the diagnosis of " shell-shock ". If the surgeon has not the time to make a thorough examination for an accurate diagnosis, he should note the symptoms on the patient's history card; and send him to the base without a diagnosis. He should never suggest to the nervous patient that he may have " shell-shock " or any other condition that could keep him back of the line indefinitely; but should always encourage him, making suggestions of improvement, rather than those which may place him on the non-effective list for a long time, or even permanently.

There is now no excuse for a diagnosis of "shell-shock", and it can no longer be used to cover up haste or ignorance in diagnosing psychoneuroses among soldiers. "Shell-shock", like other mistakes and fallacies, should and will pass into "innocuous desuetude".

